# Development of a Spectrophotometric System to Detect White Striping Physiopathy in Whole Chicken Carcasses

**DOI:** 10.3390/s17051024

**Published:** 2017-05-04

**Authors:** Maria Victoria Traffano-Schiffo, Marta Castro-Giraldez, Ricardo J. Colom, Pedro J. Fito

**Affiliations:** 1 Instituto Universitario de Ingeniería de Alimentos para el Desarrollo, Universitat Politecnica de Valencia, Camino de Vera s/n, 46022 Valencia, Spain; matrasc@upv.es (M.V.T.-S.); marcasgi@upv.es (M.C.-G.); 2 Instituto de Instrumentación para Imagen Molecular, Universitat Politecnica de Valencia, Camino de Vera s/n, 46022 Valencia, Spain; rcolom@eln.upv.es

**Keywords:** white striping, poultry, dielectric spectroscopy, fatty acids, radiofrequency, microwave, spectrophotometry, dispersion, microstructure

## Abstract

Due to the high intensification of poultry production in recent years, white chicken breast striping is one of the most frequently seen myopathies. The aim of this research was to develop a spectrophotometry-based sensor to detect white striping physiopathy in chicken breast meat in whole chicken carcasses with skin. Experiments were carried out using normal and white striping breasts. In order to understand the mechanism involved in this physiopathy, the different tissues that conform each breast were analyzed. Permittivity in radiofrequency (40 Hz to 1 MHz) was measured using two different sensors; a sensor with two flat plates to analyze the whole breast with skin (NB or WSB), and a two needles with blunt-ended sensor to analyze the different surface tissues of the skinless breast. In the microwave range (500 MHz to 20 GHz), permittivity was measured as just was described for the two needles with blunt-ended sensor. Moreover, fatty acids composition was determined by calorimetry techniques from −40 °C to 50 °C at 5 °C/min after previously freeze-drying the samples, and pH, microstructure by Cryo-SEM and binocular loupe structure were also analyzed. The results showed that the white striping physiopathy consists of the partial breakdown of the pectoral muscle causing an increase in fatty acids, reducing the quality of the meat. It was possible to detect white striping physiopathy in chicken carcasses with skin using spectrophotometry of radiofrequency spectra.

## 1. Introduction

In recent decades, the overall consumption of chicken and turkey meat has increased considerably and it is expected that in the coming years, chicken will become the most produced type of meat in the world. It has been estimated that in 2020, global chicken meat production will be around 122.5 million tons [1]. The two main reasons that are driving the success of poultry meat are the low price and the healthy nutritional profile compared to pork and beef.

As a result of increasing demand, there has been an intensification in poultry production, where modern hybrid broilers show a pectoral development greater than 20% of the body weight [2]. This production intensification has increased the incidence of abnormalities in the pectoralis muscle [3,4,5]. The most common malformations are deep pectoral myopathy [6,7] and white striping [8,9].

White striping disease is a serious, emerging issue characterized by the appearance of white stripes (WS) parallel to the muscle fiber on the surface of the *pectoralis major* muscle [10]. Some authors have classified the incidence into three categories, according to the intensity and thickness of the WS: NORMAL when the breast is not affected, MODERATE when the thickness of the WS is less than 1 mm and SEVERE when the WS covers most of the surface and the thickness is greater than 1 mm [11,12].

The presence of WS on the surface of chicken breasts affects the visual appearance of the product and decreases the degree of consumer acceptance [11]. However, the negative visual impact is not the only problem; white striping also affects the chemical composition: protein content decreases as the degree of affection increases, while there is an opposite trend in fat content, thus the presence of this hypertrophy decreases the nutritional value of the meat [13].

For this reason, white striping breasts (WSB) should not be commercialized as normal breast (NB), and should be used in the formulation of meat products such as sausages and nuggets, however, when the chicken is sold whole, the presence of the skin means that it is not possible to see the effects of this disorder. Considering this issue, it is necessary to find a reliable, efficient industrial system to detect the presence of white striping; for this reason, non-invasive sensors based on spectrophotometry at range of radiofrequency and microwaves could be a good tool to meet this challenge.

Dielectric properties expressed as permittivity can be explained as vector, polar or complex numbers. As a complex number, permittivity (ε) is composed of two terms, the dielectric constant ε′ and the loss factor ε″, which are the real and imaginary terms of permittivity, respectively. The dielectric constant is related to the tissue’s ability to absorb and store electric energy, and the loss factor is related to the dissipation of the electric energy into other energies such as thermal or mechanical energies.

In the radiofrequency and microwave range, the interaction of the photon flux with biological tissue produces three main dispersions: α, β, and γ [14]. In particular, α-dispersion (from a few Hz to a few kHz), also called the counterion effect, is induced by the orientation of mobile charges in a dielectric medium [15]. β-Dispersion (from kHz to tens of MHz) is related to the orientation of fixed charges in macromolecules such as proteins [16]. At higher frequencies of the radiofrequency range, β-dispersion results due to the surface tension charges, this phenomenon is called the Maxwell-Wagner effect. Finally, in the microwave range, γ-dispersion occurs at GHz frequencies and is due to the orientation and induction of dipolar molecules such as water [17,18,19]. Another important effect in the microwave range is ionic conductivity. It only affects the loss factor, as it produces a repulsion of charged molecules, transforming electric energy into other types [20].

The usefulness of spectrophotometry at low frequencies in the food industry has been demonstrated as a monitoring technique in a wide range of the permittivity spectra: radiofrequency, microwave and infrared (from Hz to THz). Talens et al. [20] developed a dielectric isotherm technique capable of predicting the water activity in dried orange peel using the ε′ at 20 GHz (microwave range); Shang, Guo, and Nelson [21] identified the apple varieties in the radiofrequency range. Castro-Giráldez et al. [22] demonstrated the usefulness of two dielectric ageing indexes at different frequencies (140 Hz, 500 Hz and 300 kHz) to determine pork meat ageing. Also, Trabelsi and Roelvink [23] and Damez et al. [24] demonstrated that dielectric spectroscopy is able to predict chicken and beef ageing using microwave and radiofrequency ranges, respectively. Finally, Cuibus et al. [25] and Traffano-Schiffo et al. [26] demonstrated that infrared is a good non-destructive technique to monitor freezing and drying processes.

The aim of this research was to develop a sensor based in spectrophotometry to detect white striping physiopathy in chicken breast meat in whole chicken carcass with skin.

## 2. Experimental

### 2.1. Raw Material

The experiments were carried out using boneless broiler breasts (*pectoralis major*) obtained from the SADA Group slaughterhouse located in Rafelbunyol, Valencia, Spain. After slaughter, male broilers of 42 d were bled, plucked, tempered in a cooling tunnel at 4 °C for 3 h and finally dismembered. Breasts were classified by the quality experts of the slaughterhouse plant, according to the appearance of severe WS (WSB) or NB. The selected breasts (including the skin) were transported using an isothermal bag with ice blocks to the laboratory of the Institute of Food Engineering for Development (IuIAD) at the Polytechnic University of Valencia (UPV). The samples were measured at 12 h post-mortem and maintained at 2 °C during the experimental procedure.

### 2.2. Experimental Procedure

Experiments were carried out using 40 chicken breasts: 20 were classified as NB and 20 as WSB. Prior to the dielectric measures, pH analyses of the samples were carried out using a punch pH-meter S-20 SevenEasy^TM^ (Mettler Toledo, Barcelona, Spain) to characterize the samples according to their quality. Permittivity of the samples was measured in the surface of the breasts (ventral side) in radiofrequency and microwave ranges (non-destructive measurements). In radiofrequency range, firstly, a sensor of two flat plates with circular surfaces was used to measure the permittivity of whole breast with skin (NB or WSB). After that, a two needles with blunt-ended sensor was used to measure the permittivity of each tissue of the skinless breast (muscle and adipose tissues of NB and WSB, and white stripes of WSB). In the microwave range, permittivity was measured as described above for the two needles with blunt-ended sensor. Subsequently, with the aim of analyzing the state of the fatty acids, a differential scanning calorimetry (DSC) study was performed by triplicate for samples of the ventral portion of NB and WSB. Finally, a macro and microstructural study of NB and WSB was performed using a binocular loupe and a low-temperature scanning electron microscope, respectively.

### 2.3. Structural Analysis

#### 2.3.1. Low-Temperature Scanning Electron Microscopy (Cryo-SEM)

The microstructure of NB and WSB was analyzed by Cryo-SEM. A Cryostage CT-1500C unit (Oxford Instruments, Witney, UK), coupled to a JSM-5410 scanning electron microscope (Jeol, Tokyo, Japan), was used. The sample was immersed in N_2_ slush (−210 °C) and then quickly transferred to the Cryostage at 1 kPa where sample fracture took place. Sublimation (etching) was carried out at −95 °C. The final point was determined by direct observation under the microscope, working at 5 kV. Then, once again in the Cryostage unit, the sample was coated with gold under vacuum (0.2 kPa) applied for 3 min, with an ionization current of 2 mA. The observation in the scanning electron microscope was carried out at 20 kV, at a working distance of 15 mm and temperature ≤ −130 °C.

#### 2.3.2. Optical Measurements

Optical measurements were made with a Leica MZ APO™ binocular loupe (Leica Microsystems, Wetzlar, Germany) with low magnification (8× to 80×) using incident light illumination (light reflected off the surface of the sample). It uses two separate optical paths with two objectives and two eyepieces to provide slightly different viewing angles for the left and right eyes. In this way it permits a three-dimensional visualization of the sample.

### 2.4. Permittivity Measurements

#### 2.4.1. Radiofrequency Range

**Figure 1:**
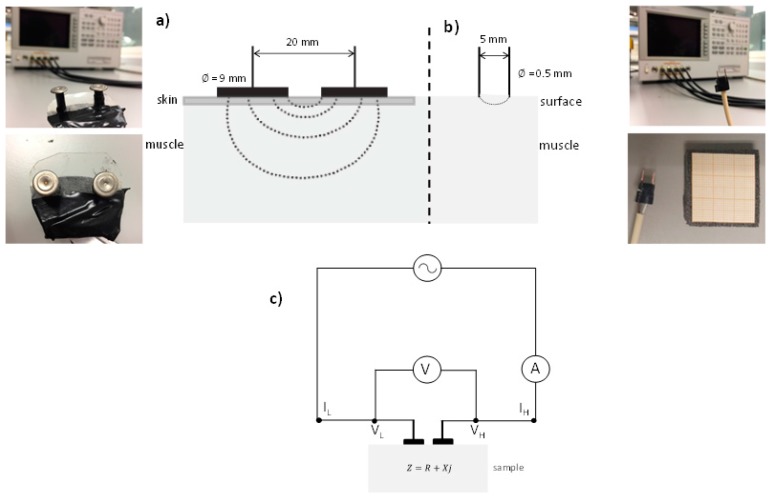
Permittivity measurement in radiofrequency, detail of each sensor: (**a**) two flat plates sensor to measure the whole breast with skin; (**b**) two needles with blunt-ended sensor to determine the permittivity in surface of the breast and (**c**) electric circuit of both sensors.

As explained above, two different sensors were used to carry out this experimental work (see Figure 1). One of them consists of two flat plates with circular surfaces sensor (Figure 1a) and it was used to measure the whole breast with skin (NB or WSB) (this configuration has high penetration depth). The other sensor consists of two needles with blunt-ended (Figure 1b) and it was used to measure the different tissues of surface breast (this configuration has low penetration depth): muscle and adipose tissues in NB and WSB, and WS in WSB. In case of the two needles sensor, its size was small enough to touch the specific tissue, and its penetration depth was low enough just to include the specific tissue. In WS and in adipose tissue, the globular conformation of the fat adipocytes, similar in the three axes, does not affect to the displacement of photon flux. However, in case of muscle, the mobility of liquid phase in the direction of the fibers changes the displacement of photon flux if it is compared with the perpendicular direction. Therefore, it was measured in perpendicular direction of fibers. It is important to highlight that the muscle tissue of WSB is referred to the muscle between the WS.

Both sensors were developed by The Institute of Food Engineering for Development (IuIAD) and The Institute for Molecular Imaging Technologies (I3M), both at the Polytechnic University of Valencia. The sensors were connected to an 4694A impedance analyzer (Agilent, Santa Clara, CA, USA). Permittivity were estimated using Equations (1)–(3). The frequency range measured was from 40 Hz to 1 MHz. Calibration of the equipment was performed in open (air) and short-circuit.

The signal obtained by the Agilent analyser is the impedance Z, and taking into account that the impedance as a complex number is $\bar{Z}=R\;+\;jX$, where the real part of the impedance is the resistance R and the imaginary part is the reactance X. The parameters *R* and *X* were transformed in ε′, ε″ as follows:

(1) $$\varepsilon^{\prime }=\;\frac{-X}{\left(R^{2}+\;X^{2}\right)}\;\frac{1}{2\pi C_{0}}$$

(2) $$\varepsilon^{\prime \prime }=\;\frac{R}{R^{2}+\;X^{2}}\;\frac{1}{2\pi fC_{0}}$$

(3) $$C_{0}=\;\frac{\varepsilon_{0}\;S}{d}$$

where *f* is the frequency (Hz), $C_{0}$ is the capacitance in the vacuum (F), *S* is the surface of the electrodes (m^2^), $\varepsilon_{0}$ is the vacuum permittivity (F/m) and d is the separation between the electrodes with differential tension (V_H_ − V_L_) (m).

#### 2.4.2. Microwave Range

Permittivity of the samples in the microwave range was measured with an Agilent 85070E open-ended coaxial probe connected to an Agilent E8362B Vector Network Analyzer. The system was calibrated using three different types of loads: open (air), short-circuit and 4 °C Milli^®^-Q water. Once the calibration was carried out, 4 °C Milli^®^-Q water was measured again to check calibration suitability. All determinations were made from 500 MHz to 20 GHz. All permittivity measurements were performed in triplicate.

### 2.5. Differential Scanning Calorimetry (DSC)

The thermal transitions of the fatty acids was performed according to the method proposed by Benedito et al. [27] using a Mettler Toledo DSC 1 differential scanning calorimeter (Mettler Toledo, Barcelona, Spain) provided with a full range temperature sensor FRS5. The calibration of the equipment was performed with the automatic calibration function FlexCal supplied by the manufacturers. Samples taken from the surface of tissue (around 22–25 mg) were introduced into aluminium pans (Mettler Toledo, ME-00026763). Water was removed by lyophilisation for 48 h using a lyophiliser Telstar Lyoalfa-6 (Telstar, Dewsbury, U.K.) as it can interfere with the fat melting in the DSC curve. The pans were hermetically sealed and an empty one was used as the reference sample. Before obtaining the DSC curves, the samples were tempered at 60 °C for 5 min, cooled to −40 °C at 10 °C/min and held at −40 °C for 5 min. The DSC curves were obtained by heating the sample from −40 °C to 50 °C at a rate of 5 °C/min.

### 2.6. Statistical Analysis

The statistical analysis was carried out with the Statgraphics Centurion XVI software (Statgraphics, Warrenton, VA, USA). One-Way ANOVA analyses were made in order to find statistically significant differences between the parameters studied for the different samples. The logistic model of modified Gompertz was fitted by using nonlinear regression.

## 3. Results and Discussion

**Figure 2:**
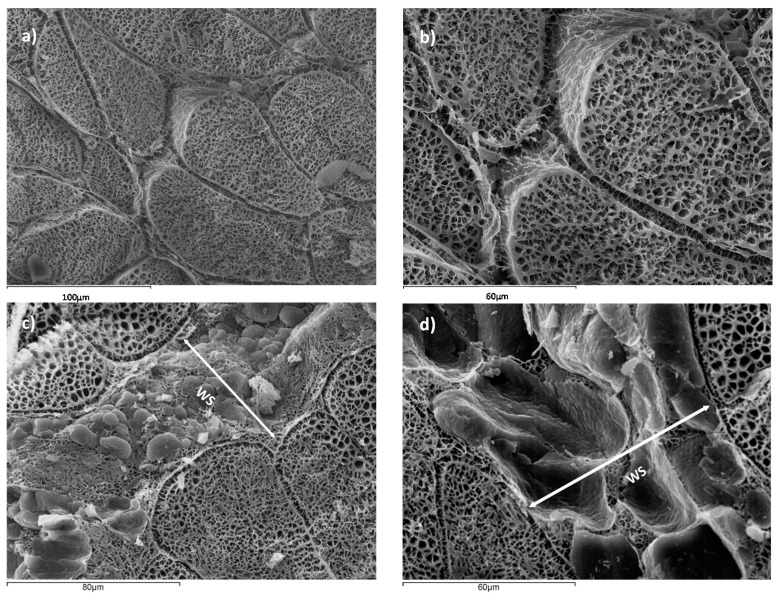
Micrographs of normal breast (NB) (**a**) 500×; (**b**) 1000× and breast affected by white striping (WSB), with the detail of the white stripe (WS) (**c**) 750× and (**d**) 1000× at 12 h post-mortem.

All the samples presented a pH value between 5.7 and 6.1, which were classified as NORMAL according to the Zhang and Barbut [28] classification. In Figure 2, a microstructural analysis of NB and WSB samples is shown. Figure 2a,b correspond to NB, where muscle tissue with correct packaging of the myofibrils is observed. However, in Figure 2c,d, it is possible to observe the deposition of adipocytes, conforming a new adipose tissue surrounded by muscle tissue, defined as white stripe (WS). This deposition of adipocytes is produced in areas which have suffered muscle breakdown. Other authors suggested that there is also an accumulation of collagen in the WS [13,29].

**Figure 3:**
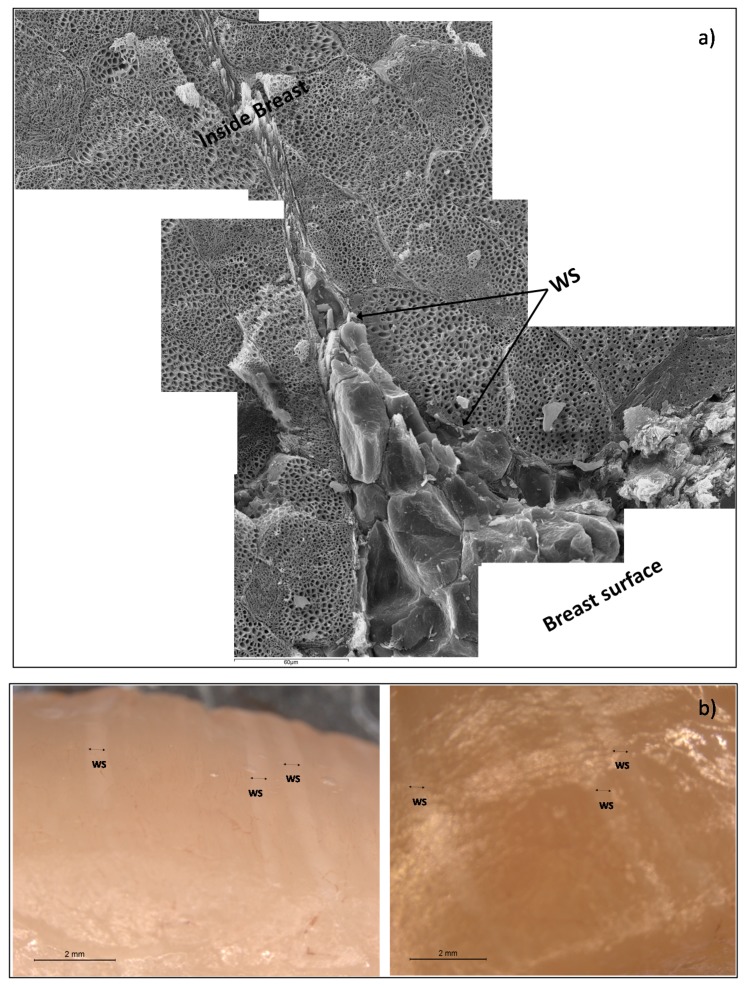
(**a**) Micrographs showing the detail of a white stripe (WS) (1000×); (**b**) binocular microscope images of WS in surface breast samples (8×).

Figure 3 shows a detail of WS in chicken breast, where a muscular rupture, from the surface breast to the internal muscle, can be appreciated, induced by forces perpendicular to the fissure, and filled with fat tissue, in order to maintain the breast integrity. Thus, the accelerated muscular growth induced by pectoral hypertrophy has caused a partial muscular rupture and the deposition of adipose tissue in this area.

Adipose tissue maintains the cohesion between muscle fibers and the elasticity of muscle tissue and therefore its activity of contraction-relaxation, conferring to the muscle the ability to transmit mechanical tension.

In addition, it is possible to appreciate that tissue breakdown is generated from the outside to the inside, so the quantity of adipose tissue is higher at the surface of the breast than the interior. Major muscle breakdown is in the external surface because the maximum tension is caused by the pectoral activity of poultry during flapping. This phenomenon produces a *pectoralis major* expansion, being maximum in the surface (reaching in this area the breakdown tension level).

Compared to NB, the WSB contains higher quantities of fatty acids, which cause the diminution of the protein content [30]. In particular, collagen increases due to hypertrophy as it is necessary to maintain the muscle structure, however, sarcoplasmic and myofibrillar proteins decrease [13].

**Figure 4:**
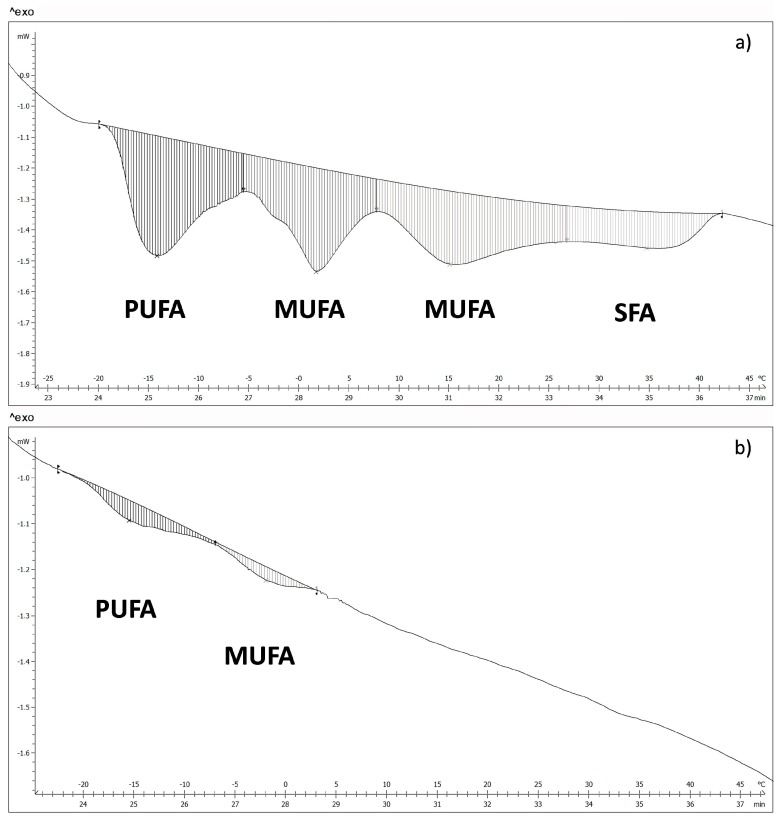
DSC thermogram of (**a**) white striping breast and (**b**) normal breast.

The fatty acids of WSB and NB, in the surface, were measured by differential scanning calorimetry. The thermograms obtained show four transitions for the WSB (Figure 4a). According to the melting temperatures, it was possible to relate each transition to the different groups of fatty acids [31,32], where polyunsaturated fatty acids (PUFA) show a melting point in the range between −17 °C and −5 °C; monounsaturated fatty acids (MUFA) between −5 °C and 27 °C and saturated fatty acids (SFA) between 27 °C and 40 °C.

Figure 4b shows a thermogram corresponding to a NB, where it is possible to appreciate that there are only two transition peaks associated with PUFA and MUFA, which suggests that there is a big difference in fatty acids content between NB and WSB. Energy values and the temperature of the different transitions for both groups of samples are shown in Table 1 and Table 2, respectively.

According to Kuttappan et al. [31] and Knothe and Dunn [32], the majority of fatty acids per groups are PUFA: linoleic acid (18:2n-6), MUFA: palmitoleic (16:1c) and oleic acids (18:1c), and finally SFA: palmitic acid (16:0). The transition temperatures of these fatty acids correspond to the temperatures 1st, 2nd, 3rd and 4th, respectively, from Table 1 and Table 2.

Mass fraction of fatty acids of NB and WSB (Table 3) were obtained from the transition energies and the latent heat of fusion of the major fatty acids in each transition [32] according to Equation (4):

(4) $$x_{fa}=\frac{E}{\Delta H^{f}}$$

Being $x_{fa}$ the mass fraction of fatty acids (kg/kg), *E* the transition energy of specific fatty acids group (J/g) and the Δ*H^f^* the latent heat of fusion of specific fatty acid (J/g).

As can be appreciated in the table, WSB show a much higher total fatty acid content than NB.

The fatty acid profile also differs between NB and WSB. WSB showed approximately 5% SFA, 23% PUFA and a 72% MUFA (expressed in relation to total fatty acids); while samples NB showed 76% PUFA, 24% MUFA and no presence of SFA (expressed in relation to total fatty acids). The change in the fatty acid profile of WSB is very important, mainly due to the presence of saturated fatty acids, principally palmitic acid. Numerous studies have shown that the consumption of saturated fat increases blood cholesterol levels, especially the LDL fraction [35,36,37]. Furthermore, the presence of WS produces, in tissue surface, an increase in total fatty acid content from 0.54% to 10.1%, so the chicken breast can no longer be considered a totally lean meat.

Permittivity was measured with different sensors. A small sensor with two needles with blunt-ended was used at radiofrequency and a coaxial probe was used in the microwave range in order to characterize the dielectric properties of the different parts of breast: muscle and adipose tissues, in NB and WSB, and WS in WSB.

One of the main problems to fit the full spectrum of radiofrequency and microwave is the appearance of three dispersions on a very large frequency range with sigmoidal shape. The Debye model is a physic model that explains the electric behaviors in this specific frequency range. This model uses different parameters to define each relaxation [38], but it is tedious to handle and difficult to fit with statistic tools. Others authors have used different math models trying to approach the Debye model [39,40]. A powerful sigmoidal model used in biological systems is the Gompertz model [41].

The dispersions shown in the dielectric constant spectra, α, β and γ, are similar to the sigmoidal models aforementioned. Therefore, dielectric constant (ε′) was modelled adjusting the experimental data using an own adaptation of the modified Gompertz model (Equation (5)) in order to obtain information on each of the dispersions:

(5) $$l\varepsilon \prime (\omega )=l\varepsilon \prime_{\infty }+\sum_{n=1}^{3}\frac{\Delta l\prime \varepsilon_{n}}{1+e^{\left((l\omega^{2}-l{\varpi_{\tau }}^{2})*\alpha_{n}\right)}}$$

where $l\varepsilon^{\prime }$ represents the decimal logarithm of the dielectric constant, $l{\varepsilon^{\prime }}_{\infty }$ the logarithm of the dielectric constant at high frequencies, lω represents the decimal logarithm of the angular velocity (obtained from the frequency), $\Delta l{\varepsilon^{\prime }}_{n}$ ($\Delta l{\varepsilon^{\prime }}_{n}$ = $\log\varepsilon \prime_{n}-\log{\varepsilon^{\prime }}_{n-1})$ the magnitude of the dispersion, $l\omega_{t}$ the logarithm of the angular velocity at relaxation time for each dispersion *n*, and $\alpha_{n}$ are the dispersion slopes.

**Figure 5:**
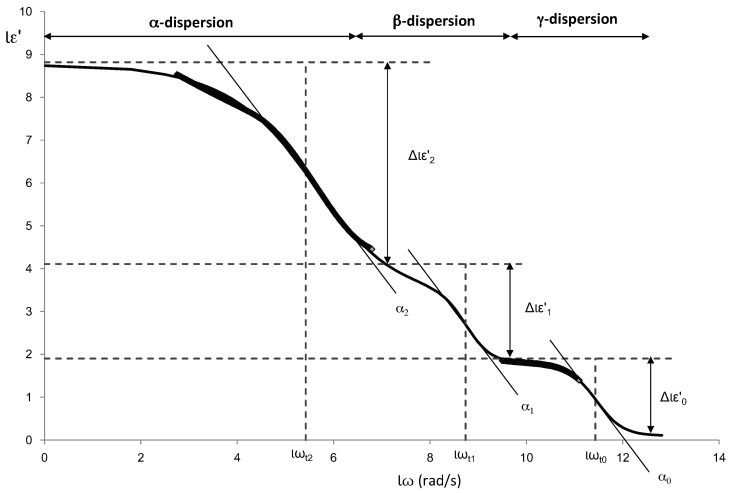
The adapted modified Gompertz model application, where the black line corresponds to the values of the mathematical model and the black diamonds are the measured data of normal breast.

Figure 5 shows an example of the different dispersions that have been modelled. In the figure the parameters of the Gompertz model are also represented.

From the Gompertz parameters, it is possible to determine the relaxation frequencies and dielectric constants of each relaxation (Equations (6)–(9)):

(6) $$\varepsilon \prime_{\alpha }=10^{\left(l\varepsilon \prime_{\infty }+\Delta l\varepsilon \prime_{\gamma }+\Delta l\varepsilon \prime_{\beta }+\frac{\Delta l\varepsilon \prime_{\alpha }}{2}\right)}$$

(7) $$\varepsilon \prime_{\beta }=10^{\left(l\varepsilon \prime_{\infty }+\Delta l\varepsilon \prime_{\gamma }+\frac{\Delta l\varepsilon \prime_{\beta }}{2}\right)}$$

(8) $$\varepsilon \prime_{\gamma }=10^{\left(l\varepsilon \prime_{\infty }+\frac{\Delta l\varepsilon \prime_{\gamma }}{2}\right)}$$

(9) $$f_{i}=10^{\frac{l\varpi_{\tau i}}{2\cdot \pi }}$$

Being i for Equation (9) each dispersion (α, β and γ). Figure 5 shows an example of radiofrequency and microwave spectra for each tissue. From the Gompertz adjustment and using Equations (6)–(9), it was possible to obtain the relaxation dielectric constants and the relaxation frequencies for each dispersion (Table 4 and Table 5).

**Figure 6:**
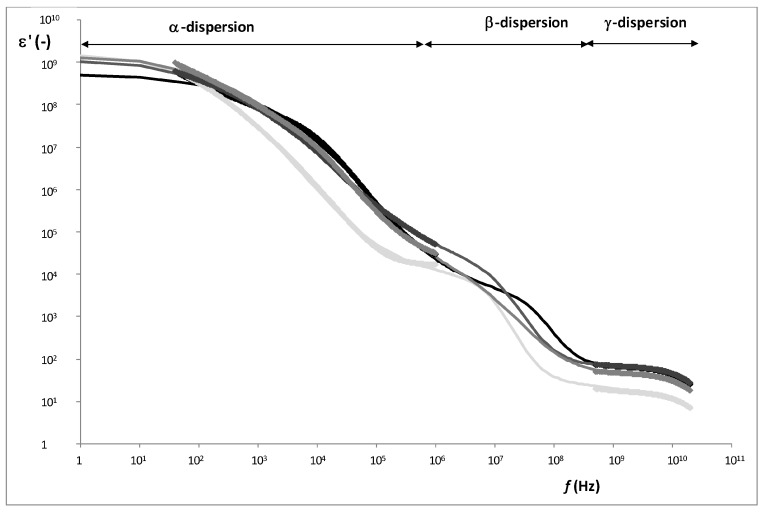
Dielectric constant spectra in radiofrequency and microwave ranges of the different tissues of white striping breast (WSB) and normal breast (NB). Where, the lines correspond to Gompertz model and the diamonds to the experimental data for each tissue.

As Figure 6 shows, three relaxations per tissue can be appreciated. In α-dispersion, the relaxation dielectric constant of muscles tissues (WSB and NB) shows a significant higher value (*p* < 0.05) than the fatty tissues (WS and adipose). The main mobile charges in muscle tissue are Ca^2+^, K^+^, Na^+^ and Mg^2+^, which have different functions, for example as the second messenger of ATP signaling; these electrolytes are solved in liquid phase, and their mobility is high. However, in fatty tissues, the electrolytes suffer the high attraction of surface tension of fatty globules, thus electrolytes maintain their orientation ability although this ability is reduced. Moreover, the white stripe sample shows a significantly higher value (*p* < 0.05) than the adipose tissue because white stripe has an accumulation of electrolytes in the interface between the partially breakdown muscle tissue and the fat globules deposited in this fissure (Table 4), where the MUFA predominance increases the adsorption of electrolytes. In order to understand the nature and interaction of the electrolytes responsible of α dispersion, the relaxation frequencies were analyzed (Table 5). The fatty tissues show the electrolytes in the same state (spin orientation); nevertheless, in case of muscle tissues, the muscle in WSB shows a similar state of electrolytes as fatty tissues. This could be due to the effect of the partial breakdown of muscle in WSB, where the nature of the electrolytes associated with the interface between fat globules and muscle tissue is different from the electrolytes in the liquid phase. This is shown clearly in the very significant difference (*p* < 0.001) of the relaxation frequency (state of electrolytes) between the muscle tissue in NB (muscle fibers without fat interactions) and the muscle in WSB.

With regard to β-relaxation, the dielectric constant of muscles tissues (WSB and NB) shows a significant higher value (*p* < 0.05) than the fatty tissues (WS and adipose), being also significant (*p* < 0.05) the difference between WS and adipose tissue.

The β-relaxation phenomena are explained by the orientation of fixed charges of the dielectric media. In muscle tissue, the structural proteins have active sites, fixed charges with orientation capacity; moreover, the main of these charges with orientation capacity are the charges involved in the actin-myosin complex [42]. This phenomenon is produced in the low MHz range as Table 5 shows, where muscles tissues have 8 ± 4 and 13 ± 5 MHz, for WSB and NB, respectively, without significant differences. Nevertheless, in the high MHz range another phenomenon occurs which is associated to the surface tension, this phenomenon is called the Maxwell-Wagner effect [15,43], and produces less absorption of energy than the effect of the active sites of proteins. In case of adipose tissue, as the relaxation frequency shows (110 ± 7 MHz), the high surface tension of the fat globules is the main contributor to β-relaxation, being the relaxation dielectric constant the lowest. In the case of WS, the breakdown of the muscle tissue and the formation of the white stripes by fat globule deposition produces both effects: the breakdown of the structural proteins of muscle tissue produces a partial degradation of myosin-actin complex and new active sites with orientation capacity, and the effect of the orientation of surface charges of fat globules. Comparing the dielectric constant of muscle tissue and adipose tissue (Table 4), it is possible to observe that the orientation capacity of active protein sites produces more absorption of energy than the capacity of orientation of surface charges of the fat globules. Taking into account that the WS is composed by broken muscle tissue and fat globules, then the β-relaxation of WS should be affected by the stronger effect, which is the orientation of active sites of proteins. This theory is strengthened by observing the β-relaxation frequency in range of the effect of protein charges (10 ± 1 MHz) and an intermediate dielectric constant value between muscles tissues and adipose tissue. The partial denaturalization of myosin-actin complex, the protein breakdown and the reduction of the electric field space occupied by proteins (Figure 3) produces a reduction of the absorption energy capacity in the WS with regard to the muscles tissues, as Table 4 shows.

Finally, regarding the γ-dispersion, the dielectric constant of the muscle tissues is higher than that of the adipose tissues (Table 4). Orientation of the dipolar molecules occurs at these frequencies, the main dipolar molecule in animal tissue is water; the adipose tissue shows less interaction with water molecules than muscle tissue due to its hydrophobic character. Water molecules in adipose tissue interact with adsorbed electrolytes of the fat, while in muscle tissue the water can be in liquid phase and also adsorbed to the tissue. Therefore the relaxation frequency is larger in muscle tissue with regard to the fat tissue since water has a higher mobility in liquid phase than adsorbed in the solid matrix (Table 5).

The permittivity of NB and WSB samples were also measured using a sensor with two flat circular plates. This kind of sensor with a raised surface was used in order to increase the penetration and to measure the permittivity at radiofrequency through the chicken skin.

Table 6 shows the average values of the dielectric constant of α and β dispersions after adjusting the data with the adapted modified Gompertz model (Equation (5)). The ANOVA performed on the WS samples compared to the normal samples reveals that the differences in the dielectric constant are significant (*p* < 0.05). As was explained in the analysis of the different tissues, in α and β dispersion, the muscle tissue produces the major effect in the dielectric constant. The measurements made in the whole breast (including the skin) follows the same behaviors, where the NB (Table 6) behaves as the muscle of the NB (Table 4) and the WSB (Table 6) behaves as a mixture between muscle of the WSB and WS (Table 4). Therefore, using sensors in the radio frequency range, it is possible to segregate chickens with white striping physiopathy before dismembering, carrying out the measurements on whole chickens.

## 4. Conclusions

It was possible to conclude that white striping physiopathy consists of the partial rupture of the pectoral muscle, possibly associated with the overgrowth process of hypertrophic breeds; the organism solves this rupture of the tissue by depositing fat globules in the break area in order to maintain the functionality of contraction-relaxation of the muscular system. The fat content and the relationship between types of fatty acids vary when white striping occurs. The change in fat content is remarkable, rising from 0.5% to 10.1%, exhibiting a higher content of MUFA and SFA fatty acids; thus, WSB cannot be considered to be a healthy food with low saturated fats. Finally, it was possible to detect white striping physiopathy in chicken carcasses with skin using sensors with flat circular surfaces in radiofrequency spectra.

## Figures and Tables

**Table 1:** Energy (J/g) and temperatures (°C) values of the fatty acids transitions of the white striping samples. Where: *T*_0_ corresponds to the initial transition temperature, *T_p_* peak transition temperature and *T_f_* the final transition temperature.

	1st Transition	2nd Transition	3rd Transition	4th Transition
***E* (J/g)**	3.3 ± 1.3	2.9 ± 1.3	3.8 ± 1.9	1.3 ± 0.5
***T*_0_ (°C)**	17 ± 2	4.6 ± 1.7	5 ± 3	27 ± 6
***T_P_* (°C)**	12.6 ± 1.6	0.9 ± 1.8	13.68 ± 0.96	35.0 ± 1.9
***T_f_* (°C)**	5 ± 2	4 ± 3	27 ± 5	39.9 ± 1.4

**Table 2:** Energy (J/g) and temperatures (°C) values of the fatty acids transitions of the normal samples. Where: *T*_0_ corresponds to the initial transition temperature, *T_p_* peak transition temperature and *T_f_* the final transition temperature.

	1st Transition	2nd Transition	3rd Transition	4th Transition
***E* (J/g)**	0.57 ± 0.04	0.24 ± 0.06	-	-
***T*_0_ (°C)**	20 ± 2	7.3 ± 0.2	-	-
***T_P_* (°C)**	15.1 ± 0.4	1.5 ± 0.6	-	-
***T_f_* (°C)**	8.1 ± 1.5	3.5 ± 0.6	-	-

**Table 3:** Mass fractions of fatty acids performed by DSC in normal (NB) and white striping (WSB) samples.

Fatty Acid	Chain	Δ*H^f^* * (J/g)	NB *x_fa_* (kg/kg)	WSB *x_fa_* (kg/kg)
SFA				
Palmitic Acid	16:0	208.2	0	0.0050 ± 0.0013
MUFA				
Oleic acid	18:1c9	75.5	0	0.059 ± 0.019
Palmitoleic acid	16:1c	189.6	0.0013 ± 0.0003	0.013± 0.005
PUFA				
Linoleic acid	18:2n-6	139	0.0041 ± 0.0003	0.0235 ± 0.0091
TOTAL Fatty Acids			0.005367 ± 0.000007	0.101 ± 0.013

* Data obtained from [33,34].

**Table 4:** Relaxation dielectric constant at each dispersion (α, β and γ) of the different tissues of normal breast (NB) and white striping breast (WSB). Data obtained by the two needles with blunt-ended sensor.

Sample	*ε′*								
	α (10^6^)			β (10^2^)			γ		
Muscle tissue WSB	22	±	6 ^a^	25	±	3 ^a^	17.3	±	0.2 ^a^
White stripe WSB	13	±	6 ^b^	11	±	5 ^b^	15.5	±	0.9 ^b^
Adipose tissue WSB, NB	6	±	2 ^c^	4	±	1 ^c^	15.0	±	0.9 ^b^
Muscle tissue NB	18	±	4 ^a^	21	±	5 ^a^	17.15	±	0.14 ^a^

Different letters (^a^–^c^) indicate significant difference between values in each column with *p* < 0.05.

**Table 5:** Relaxation frequency at each dispersion (α, β and γ) of the different tissues of normal breast (NB) and white striping breast (WSB), obtained by the two needles with blunt-ended sensor.

Sample	*f*								
	α (kHz)			β (MHz)			γ (GHz)		
Muscle tissue WSB	6	±	1 ^b^	8	±	4 ^b^	45	±	3 ^a^
White stripe WSB	7	±	4 ^b^	10	±	1 ^b^	38	±	1 ^b^
Adipose tissue WSB, NB	6	±	4 ^b^	110	±	7 ^a^	34	±	2 ^c^
Muscle tissue NB	18	±	4 ^a^	13	±	5 ^b^	43	±	1 ^a^

Different letters (^a^–^c^) indicate significant difference between values in each column with *p* < 0.05.

**Table 6:** Values of dielectric constant in α and β dispersions of white striping breast (WSB) and normal breast (NB), obtained by the sensor with two flat plates with circular surfaces.

Sample	*ε′*					
	α (10^6^)			β (10^2^)		
WSB	3.1	±	0.8 ^b^	21	±	5 ^b^
NB	4.2	±	0.3 ^a^	31	±	3 ^a^

Different letters (^a^–^b^) indicate significant difference between values in each line with *p* < 0.05.
